# Supplementary material for: Combined orbital tomography study of multi-configurational molecular adsorbate systems
Source: Nat Commun. 2019 Nov 20;10:5255. doi: 10.1038/s41467-019-13254-7 (PMC6868194; doi:10.1038/s41467-019-13254-7)
Supplement: Supplementary file 1 — Supplementary Information [file 41467_2019_13254_MOESM1_ESM.pdf]

Supplementary Information

Combined orbital tomography study of multi-configurational molecular adsorbate systems

Kliuiev et al.

I. SUPPLEMENTARY DISCUSSION

Complete LEED analysis

The Ag(110) surface exhibits a mirror symmetry which is given by the plane normal to the surface through the $[1\bar{1}0]$ direction. For any structure on the surface that is not symmetric with respect to this mirror symmetry, there exists a mirrored counterpart. For this reason, the molecules are ordered in two surface lattices that are mirror symmetric with respect to the $[1\bar{1}0]$ direction of the Ag(110) surface. In addition, for all adsorption geometries that are not symmetric with respect to the $[1\bar{1}0]$ direction, there is a mirror-symmetric counterpart as well. For sake of brevity, we show only the most important combinations of AGs and lattices in the main text. The complete sets of possible molecular registries is shown in Supplementary Figures 1 (CoPyr/Ag(110)) and 2 (Pyr/Ag(110)).

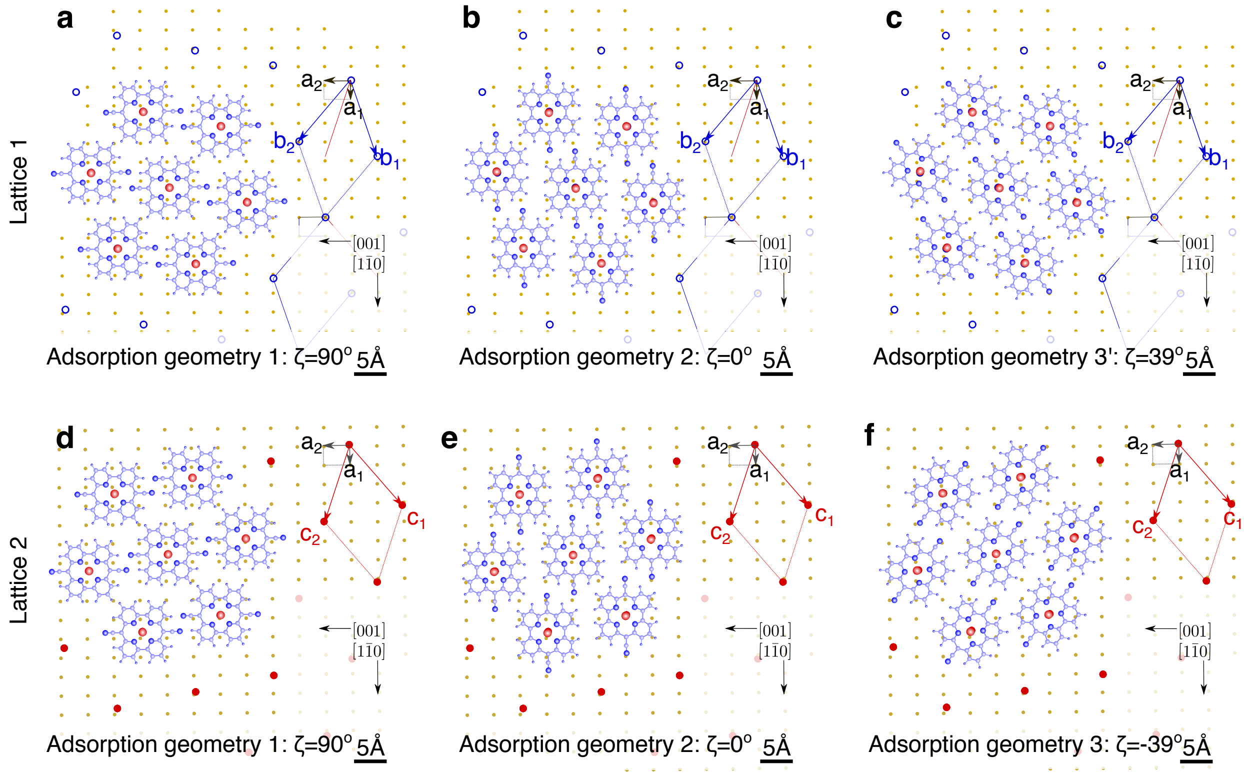

Supplementary Figure 1: Molecular registries and surface lattices of CoPyr/Ag(110) from LEED data. (a-c) Possible adsorption geometries in lattice 1. (d-f) Possible adsorption geometries in lattice 2.

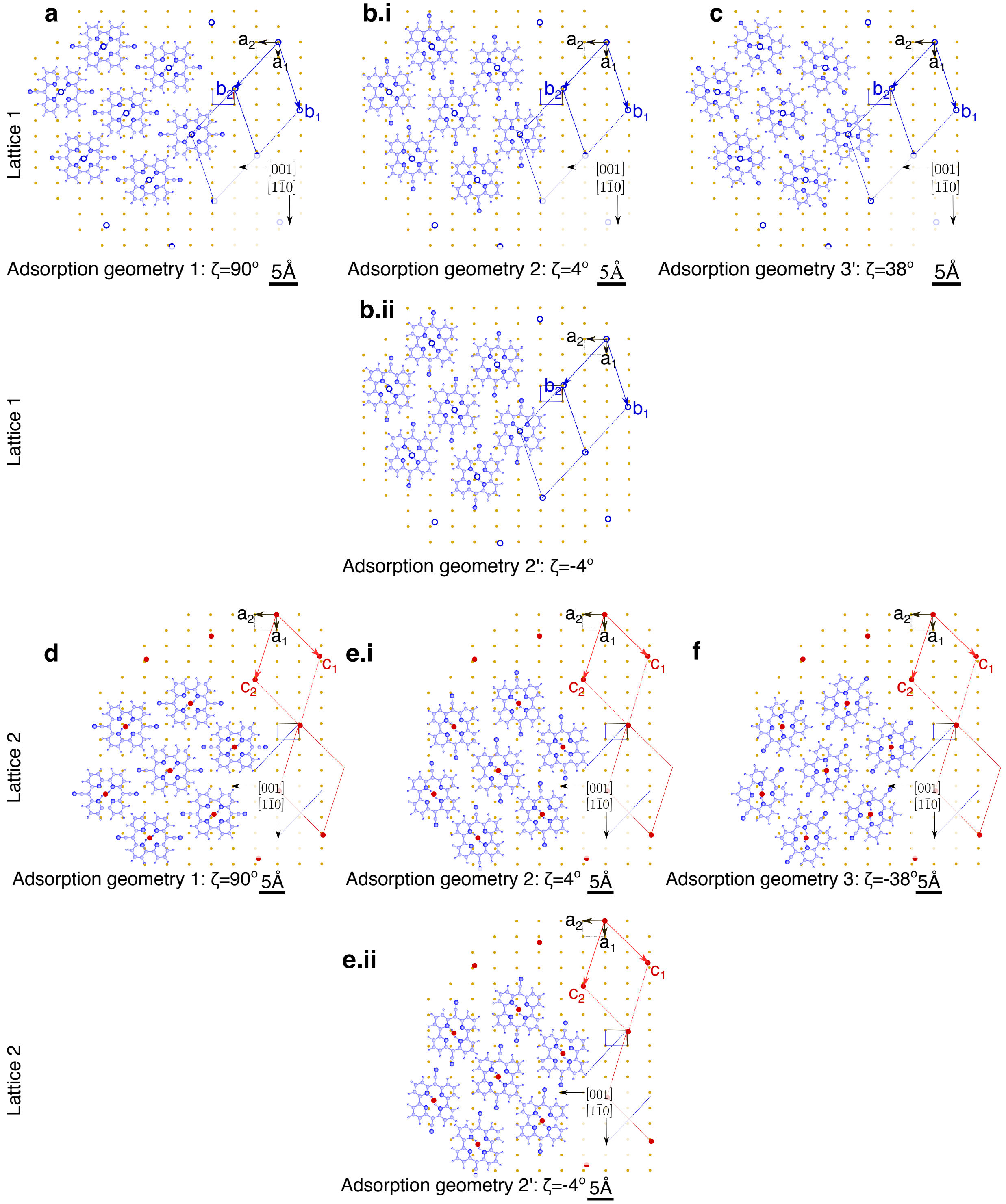

Supplementary Figure 2: Molecular registries and surface lattices of Pyr/Ag(110) from LEED data. (a-c) Possible adsorption geometries in lattice 1. (d-f) Possible adsorption geometries in lattice 2.

II. SUPPLEMENTARY METHODS

ARPES data processing

All shown PMMs were recorded at the nanoESCA endstation at the Elettra synchrotron (Trieste, Italy) [1]. Raw experimental PMMs shown in Supplementary Figure 3(a,f) are typically an average of 120 images acquired with a dwell time of 5 s for CoPyr and 1 s for Pyr, respectively. For the background subtraction, we also recorded PMMs of the clean Ag(110) surface at the respective binding energies of the molecular states (Supplementary Figure 3(b,g)). Background-subtracted PMMs are shown in Supplementary Figure 3(c,h). In order to account for matrix element effects due to the polarization of the incoming light (p-polarized, 15° grazing incidence angle), the background-subtracted data was normalized with the $|\mathbf{A} \cdot \mathbf{k}_\mathrm{f}|^2$ factor, where $\mathbf{A}$ is the vector potential of the incoming light and $\mathbf{k}_\mathrm{f}$ is the photoelectron momentum (Supplementary Figure 3(d,i)). Finally, the data was symmetrized with respect to the geometrical center to increase the contrast and thus facilitate comparison with the simulated data (Supplementary Figure 3(e,j)).

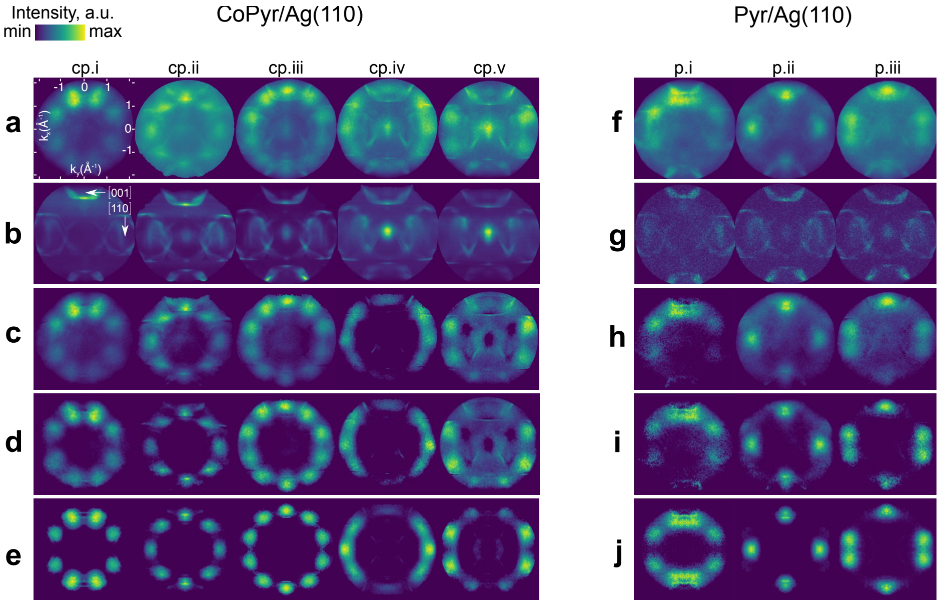

Supplementary Figure 3: ARPES data processing. (a,f) Raw experimental PMM. (b,g) PMM of Ag(110) substrate background at binding energy of the indicated molecular state. (c,h) PMM after subtraction of the Ag(110) background. (d,i) PMM after normalization with the $|\boldsymbol{A} \cdot \boldsymbol{k}_\mathrm{f}|^2$ factor. (e,j) PMM symmetrized with respect to the geometrical center.

Simulation of PMM from DFT data

The simulation of PMMs from DFT data is based on our previously published procedure [2]. In brief, the 3D DFT orbital distributions were centered in the computational domain. They were zero-padded to N=512 pixels in each dimension. 3D Fourier transform of zero-padded orbital distributions delivered 3D distributions in momentum space. In the experiment, PMMs were recorded at fixed electron kinetic energies. Because optical transitions are direct transitions in reciprocal space, the momenta in the PMMs are sampled on a hemisphere with radius k_0 set by the photoelectron kinetic energy. In 3D distributions in momentum space, all pixel values in the range of $[0.95k_0, 1.05k_0]$ were kept, while others were set to 0, thus yielding 2D distributions in momentum space. To account for the finite numerical aperture of the system, the values of all pixes lying outside the ± 2 Å^{-1} range were set to 0. Finally, the resulting 2D distributions in momentum space were squared to yield simulated PMM intensity distributions.

Orbital density distributions retrieved from DFT calculations in vacuum and on the Ag(110) surface were treated in the same way. In addition to the gas phase DFT simulations shown in Figure 4 of the main text, we show the same data for the simulations of CoPyr and Pyr on the Ag(110) cluster in Supplementary Figure 4. Hardly any difference can be discerned between the simulations in vacuum and of the molecules on the Ag surface. This further confirms the robustness of the molecular wave functions.

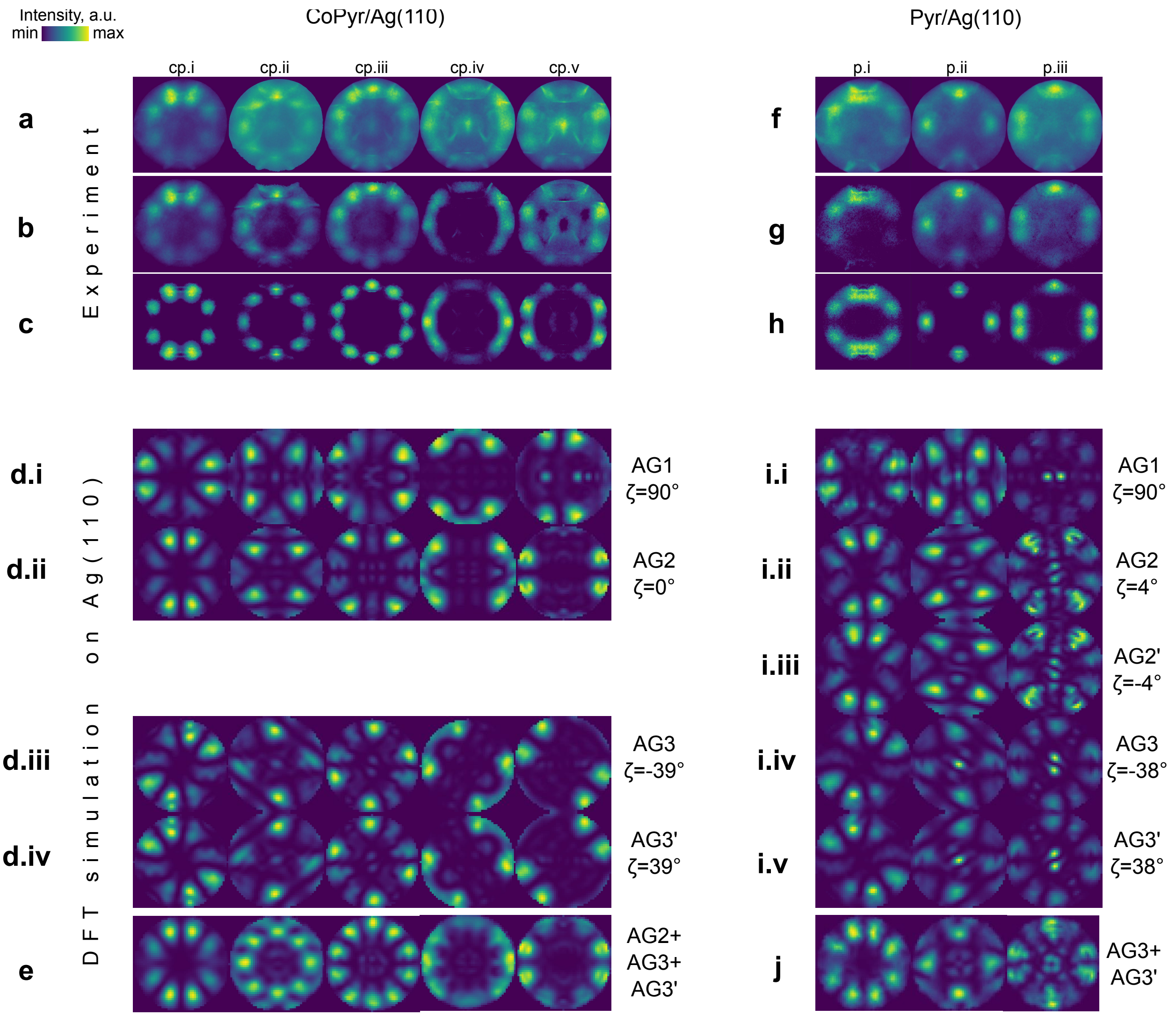

Supplementary Figure 4: APRES data and simulations of CoPyr (a-e) and Pyr (f-j) on Ag(110). (a,f) Raw ARPES data. (b,g) PMMs after background subtraction and (c,h) after normalisation. (d,i) Simulated PMMs for indicated adsorption geometries. (e,j) Incoherent sum of PMMs: CoPyr (AGs 2, 3, 3' weighted 1.5:1:1), Pyr (AGs 3, 3' weighted 1:1).

III. SUPPLEMENTARY TABLES

Orbital correspondence tables

As discussed in the main text, DFT does not necessarily reproduce the correct orbital hierarchy of complex molecular systems. In Supplementary Tables 1 and 2 we show the correspondence between experimentally determined molecular states and the molecular orbitals derived from the DFT calculations. In case of CoPyr, the simple gas phase DFT calculation completely fails to reproduce the right hierarchy. The higher level calculations of the surface cluster render a much more consistent picture. For the empty Pyr ligand, which is eventually a relatively simple conjugated π-system, the orbital hierarchy is correctly reproduced by the simple gas phase DFT calculations.

The molecular orbitals of the high-level calculations of the adsorbate systems are ordered by computed energies. By analysis of the PDOS we could identify $mo1165$ as HOMO of CoPyr in all three adsorption geometries. In Pyr, $mo1035$ was identified as HOMO for AG1 and $mo1131$ for AGs 2 and 3.

Exp. state	Gas phase DFT	AG1	AG2	AG3
cp.i	HOMO-6	mo1106	mo1106	mo1106
cp.ii	HOMO-2	mo1127	mo1127	mo1127
cp.iii	HOMO	mo1144	mo1135	mo1135
cp.iv	HOMO-3	mo1145	mo1145	mo1145
cp.iv	LUMO+5	mo1165	mo1165	mo1165

Supplementary Table 1: Correspondence between experimentally identified molecular states from ARPES data and DFT calculations for CoPyr on Ag(110).

Exp. state	Gas phase DFT	AG1	AG2	AG3
p.i	HOMO-2	mo1099	mo1100	mo1102
p.ii	HOMO-1	mo1123	mo1123	mo1123
p.iii	HOMO	mo1035	mo1131	mo1131

Supplementary Table 2: Correspondence between experimentally identified molecular states from ARPES data and DFT calculations for Pyr on Ag(110).

IV. SUPPLEMENTARY REFERENCES

[1] Wiemann, C.; Patt, M.; Krug, I. P.; Weber, N. B.; Escher, M.; Merkel, M.; Schneider, C. M. A New Nanospectroscopy Tool with Synchrotron Radiation: NanoESCA@Elettra. *e-J. Surf. Sci. Nanotech* **2011**, *9*.

[2] Kliuiev, P.; Latychevskaia, T.; Zamborlini, G.; Jugovac, M.; Metzger, C.; Grimm, M.; Schöll, A.; Osterwalder, J.; Hengsberger, M.; Castiglioni, L. Algorithms and image formation in orbital tomography. *Phys. Rev. B* **2018**, *98*, 085426.
